# A bibliometric analysis of research on factors influencing STEM career choices: gender, identity, self-efficacy, and self-concept

**DOI:** 10.3389/fpsyg.2026.1859525

**Published:** 2026-07-30

**Authors:** Hafife Bozdemir Yüzbaşıoğlu

**Affiliations:** Department of Primary Education, Faculty of Education, Kastamonu University, Kastamonu, Türkiye

**Keywords:** bibliometric analysis, gender, identity, self-concept, self-efficacy, STEM career choice

## Abstract

This study examines a search-defined body of STEM career-choice literature retrieved through queries combining STEM-related, career-related, gender-related, and psychosocial-construct terms, including identity, self-efficacy, and self-concept. Records from Web of Science and Scopus were screened through a PRISMA-informed process, yielding 181 publications from 2001 to 2026. Bibliometric performance analysis and science-mapping techniques were conducted in R to examine publication trends, leading contributors, geographical patterns, international co-authorship, keyword co-occurrence, thematic structure, and thematic evolution. Annual scientific output generally increased, particularly after 2010, although the pattern fluctuated across years. The highest observed output was recorded in 2025, with 27 publications, while an exploratory life-cycle model estimated a peak of approximately 17 publications around 2021.9. Because the model fit was moderate and 2026 was incomplete, this trajectory was interpreted cautiously. Publication activity was geographically concentrated, and single-country publications outnumbered multiple-country publications among the leading corresponding-author countries. Keyword analysis identified two connected clusters: one emphasizing science, gender, self-efficacy, STEM, identity, achievement, and career aspirations, and another centered on career choice, math, technology, students, education, engineering, motivation, and self-concept. The thematic map positioned career choice, technology, and students as a motor theme; science, gender, and self-efficacy as a basic or transversal theme; goals, persistence, and adolescents as a niche theme; and race, qualitative research, and school students as an emerging or declining theme. Thematic evolution indicated a shift from an earlier structure involving motivation, adolescence, math, women, and science education toward a later structure centered on gender and career choice.

## 1 Introduction

In the 21st century, science, technology, engineering, and mathematics (STEM) fields are among the key drivers of the global economy and societal transformation. However, participation rates in STEM education remain limited in many countries. According to OECD (2023) data, less than one-quarter of higher education graduates receive an education in STEM fields. This situation represents not only a shortage in the labor supply but also a limitation on cognitive diversity, which is critical for innovation processes. Indeed, diverse cognitive perspectives play a significant role in collective problem-solving and innovation (Hong and Page, 2004). One of the most prominent and persistent dimensions of this overall imbalance is gender inequality. Although women constitute the majority of higher education graduates, their representation among STEM graduates is notably low (OECD, 2023). This situation has long been explained using the “leaky pipeline” metaphor, which describes how individuals drop out of STEM fields at various stages of their educational journey (Tajmel, 2019). However, it is increasingly emphasized that this linear approach fails to adequately capture the multidimensional and dynamic nature of individuals’ career trajectories (Wu and Uttal, 2020). In this context, one key finding in the literature is the inconsistency between students’ interest in STEM fields and their desire to pursue careers in them. Research indicates that while students may develop positive attitudes toward science, they may still be reluctant to choose STEM-based careers. The phrase often summarizes this situation: “Science is important, but not for me” (Jenkins and Nelson, 2005). Particularly for female students, despite their interest in and success in scientific fields, the fact that these fields do not sufficiently align with their individual identities leads STEM careers to be perceived as “less suitable for them” (Archer et al., 2013). Gendered expectations and stereotypes may shape how individuals perceive their place within STEM environments and the suitability of STEM career pathways for themselves (Makarova et al., 2019). These influences highlight the need to examine STEM career choice in connection with psychosocial constructs such as identity, self-efficacy, and self-concept. From an educational psychology perspective, STEM career choice can therefore be understood as a process shaped by the interaction between gendered experiences and individuals’ beliefs about who they are, what they are capable of accomplishing, and whether STEM is compatible with their perceived abilities and sense of self (Hazari et al., 2010; Chemers et al., 2011; Sadler et al., 2012; Wang and Degol, 2013; Chen et al., 2024; Jiang et al., 2025).

In the literature on inequalities in STEM participation, numerous explanations of the representation of women and diverse groups in STEM fields have been developed, and it is evident that this process is examined across its biological, pedagogical, social, and cultural dimensions (Blickenstaff, 2005). However, it has been observed that explanations based solely on cognitive competence or academic achievement are limited in their ability to fully account for individuals’ decision-making processes regarding STEM career choices. Indeed, research indicates that possessing sufficient competence in mathematics and science is not sufficient for individuals to choose careers in these fields (Wang and Degol, 2017). This situation has led to a shift in the research focus from external explanations toward the subjective and internalized relationships individuals establish with STEM fields (Holmegaard et al., 2014). In this context, psychological factors such as identity, self-efficacy, self-concept, together with related processes such as belonging and recognition, have received increasing attention in STEM career research (Carlone and Johnson, 2007; Hazari et al., 2010; Jiang et al., 2024; Lu et al., 2024). This growing attention reflects an effort to understand STEM career development not only through academic performance and structural opportunities but also through individuals’ perceptions of competence, sense of self, and relationships with STEM environments. In this process, gender functions not merely as a demographic variable but as an interpretive lens that shapes how individuals make sense of STEM fields (Cheryan et al., 2017). Gender norms indirectly influence career decisions by affecting individuals’ sense of belonging and level of interest (Cheryan et al., 2015). This indicates that, particularly for women, the relationship they establish with STEM fields is not solely competence-based but also a process rooted in identity and belonging (Carlone and Johnson, 2007). Therefore, STEM career development should be explained not only through opportunity structures but also through the psychosocial mechanisms that shape how individuals relate to these fields (Lent et al., 1994).

These developments highlight the need to clarify the conceptual relationships among gender, identity, self-efficacy, self-concept, and STEM career choice. The following section therefore outlines the conceptual distinctions among these constructs and positions Social Cognitive Career Theory as an interpretive framework for organizing their relationships within the search-defined body of literature examined in this study.

## 2 Conceptual and theoretical background

STEM career choice is a developmental process shaped by the interaction of educational experiences, individual beliefs, social expectations, and perceived career opportunities (Abe and Chikoko, 2020; Mansour, 2025). Although students’ academic achievement and interest are important components of this process (Wang et al., 2015; Aeschlimann et al., 2016; Blotnicky et al., 2018), their career-related interests, aspirations, and decisions are also shaped by how they interpret their competence, perceive their compatibility with STEM fields, and imagine themselves in future STEM roles (Chen et al., 2024; Wang and Degol, 2017; Grimalt-Álvaro et al., 2022). From this perspective, gender, identity, self-efficacy, and self-concept represent related but conceptually distinct dimensions through which STEM career development can be understood.

Gender is relevant to STEM career choice not only as a demographic characteristic but also through the social expectations, stereotypes, opportunities, and recognition processes associated with it. Gender stereotypes may influence whether students perceive STEM fields as personally suitable and attainable, even when they demonstrate interest or academic competence in these areas (Cheryan et al., 2015; Cheryan et al., 2017; Makarova et al., 2019). Accordingly, gender does not independently determine career choice; rather, it interacts with contextual and psychological processes that influence how individuals evaluate their relationship with STEM pathways (Wang and Degol, 2013; Godwin et al., 2016; Wang and Degol, 2017; Chan, 2022). Identity concerns the extent to which individuals see themselves—and are recognized by others—as legitimate participants in science or STEM (Hazari et al., 2010; Vincent-Ruz and Schunn, 2018). Students may perform successfully in STEM subjects but remain reluctant to pursue related careers when they do not perceive STEM as compatible with who they are (Collins et al., 2020; Miles and Naumann, 2021). Conversely, recognition by teachers, peers, and significant others may support students’ identification with STEM and make related career pathways appear more attainable (Ladachart et al., 2024). Self-efficacy refers to individuals’ beliefs in their capacity to organize and carry out the actions required to perform specific tasks successfully (Bandura, 1997). In STEM contexts, these beliefs may relate to performing mathematics-, science-, engineering-, or technology-related activities (Chemers et al., 2011; Luo et al., 2021). Within Social Cognitive Career Theory, learning experiences contribute to the development of self-efficacy and outcome expectations, which shape interests and goals and, in turn, influence academic and career choices, performance, and persistence (Lent et al., 1994; Lent et al., 2017). Self-concept refers to individuals’ broader perceptions and evaluations of their competence within an academic domain (Marsh et al., 1991; Marsh, 2007; Lee, 2009). Although self-efficacy and self-concept share a common concern with perceived competence, they represent distinct forms of self-belief: self-efficacy describes individuals’ perceived capability to complete specific tasks, whereas self-concept involves broader evaluative judgments that are shaped partly by normative and internal comparisons (Bong and Skaalvik, 2003; Marsh et al., 1991; Marsh et al., 2008). Empirical evidence indicates that stronger STEM self-concept is positively associated with higher aspirations to pursue STEM-related careers (Chen et al., 2024).

Taken together, these constructs suggest that STEM career choice cannot be adequately explained by any single academic, psychological, or contextual factor in isolation (Lent et al., 1994; Sahin et al., 2018). Rather than being understood as the product of the independent effects of these constructs, STEM career orientations can be understood as a dynamic process shaped by the interrelations among these dimensions (Byars-Winston and Rogers, 2019). Social Cognitive Career Theory provides an organizing framework for examining this multidimensional process by linking learning experiences, self-efficacy, outcome expectations, personal goals, and contextual supports and barriers with the development of career interests, choices, performance, and persistence (Lent et al., 1994; Lent and Brown, 2019). In the present study, self-efficacy retains its central position within SCCT, while gender, identity, and self-concept are considered complementary constructs representing sociocultural, identity-related, and broader competence-related dimensions of students’ orientations toward STEM (Carlone and Johnson, 2007; Parker et al., 2014; Wang and Degol, 2017). Accordingly, SCCT is used as an organizing interpretive lens for understanding how these conceptually distinct dimensions are represented and related within the search-defined corpus. It is not treated as a model empirically tested by the bibliometric analyses.

## 3 Research gap

Existing evidence syntheses and bibliometric studies have contributed substantially to understanding the development of STEM education and the factors associated with STEM participation, success, aspirations, and career choice. Bibliometric studies have mapped the broader structure of STEM education or examined diverse influences on students’ intentions, program choices, and academic success (Hinojo-Lucena et al., 2020; Maphosa et al., 2022; Kumari and Singh, 2023). Systematic and mapping reviews have organized the factors associated with STEM career development using different conceptual categories. López et al. (2023) classified these factors as environmental factors, social influencers, and personal factors, whereas Zhou and Shirazi (2025) identified self-motivation, social persuasion, self-efficacy, personal utility, and positive STEM experiences as influential themes in young people’s STEM career aspirations and choices. Shulga et al. (2023), in turn, systematically reviewed empirical research on learners’ STEM career choices and organized the literature around STEM activities, teacher-related influences, associated variables, learners’ career choices, and parental attitudes. Gender-focused review and bibliometric studies have also examined gender disparities, women’s participation, and strategies for increasing girls’ motivation and engagement in STEM (Carrión et al., 2024; Pineda et al., 2025; Camila Francisco de Castro et al., 2026). Despite these contributions, previous reviews have generally adopted either a broad factor-based perspective or a more focused emphasis on gender, participation, motivation, or academic outcomes. Consequently, limited evidence is available on the relative visibility and conceptual positioning of gender, identity, self-efficacy, and self-concept within a common body of STEM career-choice literature. Mapping these constructs within the same bibliometric corpus is valuable because they represent complementary dimensions of career development: sociocultural positioning, identification with STEM, task-specific capability beliefs, and broader evaluations of competence. Although these dimensions have been examined separately or included within wider classifications of influencing factors, their conceptual connections, thematic positions, and development over time remain insufficiently mapped within a shared analytical framework. Without such an integrated perspective, it is difficult to determine whether these constructs have developed as coherent or fragmented research strands and which relationships require further theory-driven investigation.

The present study addresses this gap by conducting a bibliometric and science-mapping analysis of publications indexed in Web of Science (WoS) and Scopus between 2001 and 2026. It provides a focused examination of the publication output, collaboration patterns, conceptual networks, thematic structure, and thematic evolution of a search-defined corpus of STEM career-choice research retrieved through queries combining STEM-related, career-related, gender-related, and psychosocial-construct terms. The psychosocial search block included identity, self-efficacy, and self-concept as alternative terms. In doing so, the study complements previous reviews by examining how these constructs are positioned within the knowledge structure of the selected literature and how their thematic visibility and connections vary across the periods examined. Because the search strategy required records to match all four conceptual search blocks, the corpus should be interpreted as a deliberately delimited, search-defined body of literature rather than as an exhaustive representation of STEM career-choice research or as evidence that all focal constructs were examined as equally central empirical variables in every publication.

## 4 Aim, research questions, and contributions

To address the identified research gaps, the present study aims to examine the structural and temporal development of a corpus of STEM career-choice research constructed through predefined search and screening criteria. The database queries combined STEM-related, psychosocial-construct, gender-related, and career-related search blocks, with identity, self-efficacy, and self-concept included as alternative terms within the psychosocial block. For this purpose, publications indexed in WoS and Scopus between 2001 and 2026 were analyzed using bibliometric performance indicators and science-mapping techniques. The study examines annual scientific production, productive authors and publication sources, geographical representation, international co-authorship, keyword relationships, thematic structure, and thematic evolution. The study maps the relative positioning, conceptual connections, and thematic development of gender, identity, self-efficacy, and self-concept within the analyzed corpus. Social Cognitive Career Theory is used as an organizing theoretical lens for interpreting these patterns rather than as a model tested or validated by the bibliometric analysis.

Based on this aim, the study addresses the following research questions:

1. What are the trends in annual scientific production within the selected corpus of STEM career-choice research?
2. Who are the most productive authors and publication sources, and which countries are most frequently represented in the analyzed corpus?
3. What patterns of international co-authorship are evident in the publications included in the corpus?
4. How is the conceptual structure of the selected literature represented through the co-occurrence network of keywords?
5. What motor, basic, niche, and emerging or declining themes are identified through thematic map analysis?
6. How does thematic evolution analysis reflect the conceptual and thematic development of the selected literature over time?

By addressing these questions, the study offers four main contributions. First, it combines records from WoS and Scopus, providing a broader source base than a single-database analysis while acknowledging that the corpus remains bounded by the selected databases and search strategy. Second, it integrates performance analysis, international co-authorship analysis, keyword co-occurrence, thematic mapping, and thematic evolution within a single analytical design. Third, it maps how gender, identity, self-efficacy, and self-concept are positioned and connected within the keyword-based conceptual structure of the selected literature and how the themes in which these concepts are represented have developed over time. Finally, by interpreting the mapped structure through an educational psychology perspective and using SCCT as an organizing lens, the study may inform future theory-driven research on how gendered experiences, identity-related processes, task-specific capability beliefs, and broader competence perceptions are associated with STEM career development.

## 5 Methods

### 5.1 Study design and reporting framework

This study was designed as a bibliometric and science-mapping review. Its aim was to map the bibliographic, conceptual, and thematic structure of a corpus of STEM career-choice research constructed through predefined search and screening criteria. The database queries combined STEM-related, psychosocial-construct, gender-related, and career-related search blocks, with identity, self-efficacy, and self-concept included as alternative terms within the psychosocial block. This search-based delimitation defined the conceptual scope of the corpus without requiring all focal concepts to be examined as equally central empirical variables in every publication. Accordingly, the review process was organized around database selection, search strategy, eligibility assessment, duplicate removal, keyword cleaning and normalization, and bibliometric/science-mapping procedures. To enhance transparency in corpus construction, a PRISMA-informed flow diagram was used to report the identification, screening, eligibility assessment, duplicate removal, and final inclusion of records. The PRISMA framework was used as a reporting tool for corpus construction. The analytical framework of the study followed bibliometric and science-mapping logic, including performance analysis, keyword co-occurrence analysis, thematic mapping, and thematic evolution analysis.

### 5.2 Data sources and search strategy

Data were retrieved from the WoS and Scopus databases. These databases were selected because they provide broad multidisciplinary coverage and detailed bibliographic metadata, including titles, abstracts, keywords, author information, source titles, affiliations, citation data, and publication years, which are required for bibliometric and science-mapping analyses. The combined use of WoS and Scopus was preferred to increase the coverage of the corpus, reduce potential single-database bias, and capture research published across educational psychology, science education, STEM education, career development, and gender-related educational research (Falagas et al., 2008; Mongeon and Paul-Hus, 2016; Kumpulainen and Seppänen, 2022; Sindhu and Gupta, 2025). The study was therefore not restricted to a single disciplinary category or journal field. Instead, it was designed to capture an interdisciplinary body of literature relevant to STEM career choice across educational psychology, science education, STEM education, career development, and gender-related educational research. No additional restriction was applied based on a specific disciplinary classification, provided that the records met the conceptual and topical eligibility criteria of the study.

The searches were conducted in the WoS database on 20 February 2026 and in the Scopus database on 26 February 2026. The WoS search was conducted using the Topic field, and the Scopus search was conducted using the TITLE-ABS-KEY field. The exact search strings were as follows:

WoS: TS = ((“STEM” OR “science education”) AND (“identity” OR “self-efficacy” OR “self-concept”) AND (“female*” OR “girl*” OR “gender”) AND (“career choice” OR “career aspiration*” OR “vocational interest”)).

Scopus: TITLE-ABS-KEY ((“STEM” OR “science education”) AND (“identity” OR “self-efficacy” OR “self-concept”) AND (“female*” OR “girl*” OR “gender”) AND (“career choice” OR “career aspiration*” OR “vocational interest”)).

The search strategy was designed to construct a deliberately delimited bibliometric corpus by combining four conceptual search blocks: a STEM-related block, a psychosocial-construct block, a gender-related block, and a career-related block. Alternative terms within each block were connected using the OR operator, while the four blocks were connected using the AND operator. Accordingly, each retrieved record was required to match at least one term from each block within the database-specific searchable fields. This retrieval condition defined the conceptual boundaries of the corpus but did not imply that all focal concepts were examined as equally central empirical variables in every publication. The terms “STEM” and “science education” were used to capture the educational and disciplinary context. The terms “career choice,” “career aspiration*,” and “vocational interest” were used to capture career-related orientations. The terms “identity,” “self-efficacy,” and “self-concept” were included as alternative terms within the psychosocial-construct block, while “female*,” “girl*,” and “gender” were used to represent the gender-related scope of the study.

The search was limited to English-language peer-reviewed journal articles published between 2001 and 2026. English-language publications were included because English is the dominant language of international academic indexing in WoS and Scopus. Journal articles were selected because they represent peer-reviewed scholarly outputs with complete bibliographic metadata suitable for bibliometric analysis. Conference papers, book chapters, editorials, and other non-article document types were excluded.

The year 2001 was selected as the starting point to capture the development of the literature in the 21st century, during which research on STEM participation, gendered educational pathways, and psychosocial constructs such as identity, self-efficacy, and self-concept became increasingly visible in relation to career choice. Records from 2026 were retained to include the most recent publications indexed at the time of data collection. However, because 2026 was an incomplete publication year when the searches were conducted, publication trends and temporal patterns involving 2026 were interpreted cautiously and were not treated as evidence of a definitive decline or long-term trend.

### 5.3 Screening and eligibility assessment

**Figure 1:**
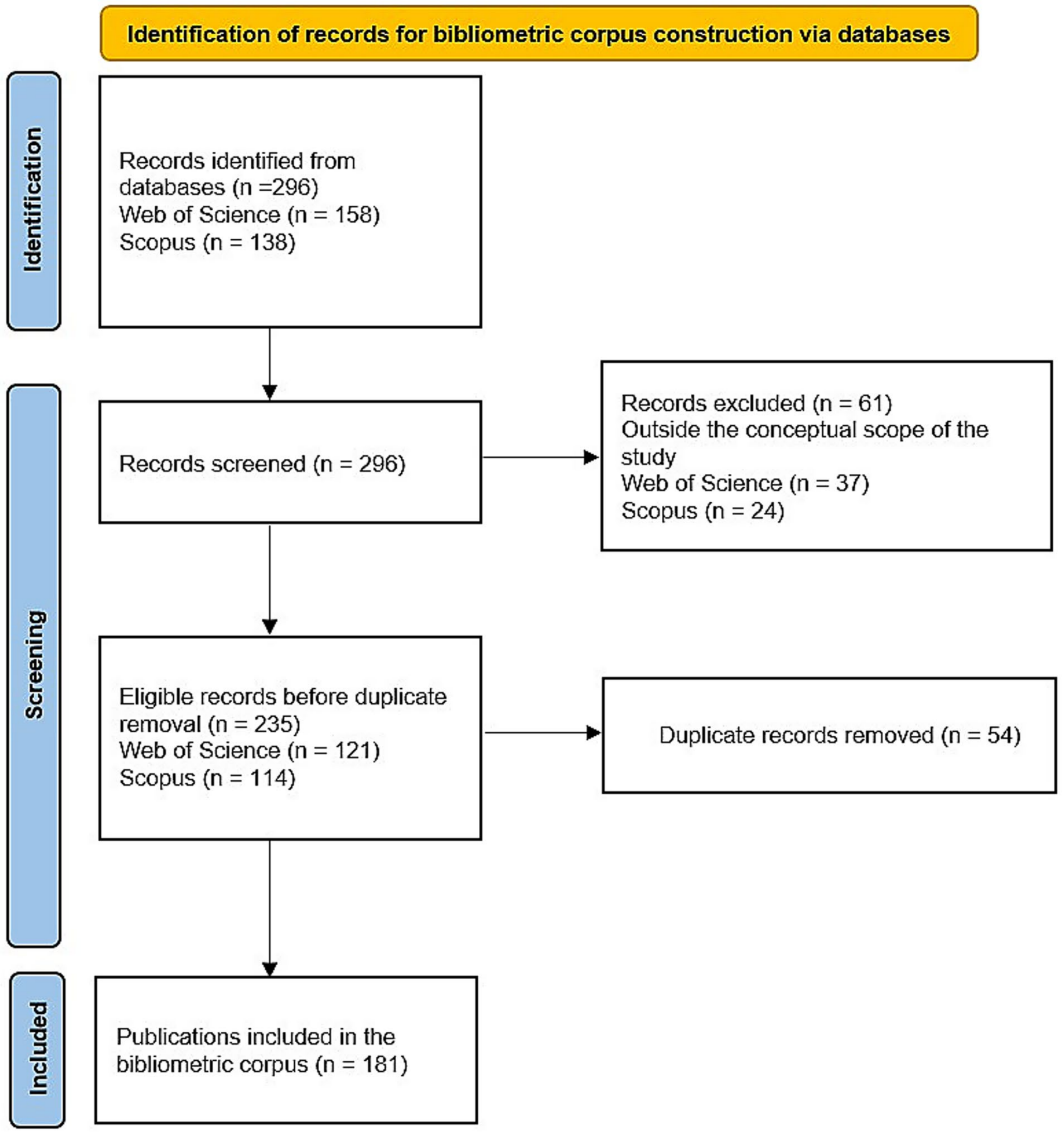
PRISMA-informed flow diagram of bibliometric corpus construction.

The corpus construction process, including record identification, screening, eligibility assessment, duplicate removal, and final inclusion, is illustrated in the PRISMA-informed flow diagram presented in Figure 1. The initial database search yielded 158 records from WoS and 138 records from Scopus, resulting in 296 records before eligibility assessment.

Figure 1 presents the PRISMA-informed flow diagram of bibliometric corpus construction (Page et al., 2021).

The retrieved records were screened according to the inclusion and exclusion criteria defined for the study. First, titles and abstracts were reviewed to determine whether the records were substantively related to STEM career choice, career aspirations, or vocational interest and were consistent with the broader conceptual scope established by the STEM-related, psychosocial-construct, gender-related, and career-related search blocks. The screening process did not require all focal concepts to be examined as equally central empirical variables in each publication. Rather, it was used to identify records in which the search terms reflected a substantively relevant STEM career-development context rather than an incidental or peripheral occurrence within the searchable fields. When the relevance of a record could not be determined from the title and abstract alone, the full text was consulted. Records that were clearly outside the scope of STEM career development, did not substantively address career choice, career aspirations, or vocational interest, or did not align with the broader conceptual boundaries established by the search strategy were excluded.

After this screening and eligibility assessment, 121 records from WoS and 114 records from Scopus were retained, resulting in 235 eligible records before duplicate removal. A total of 61 records were excluded at this stage because they did not meet the conceptual and topical eligibility criteria of the study. The eligible records obtained from the two databases were then merged into a single dataset using the R programming environment. Duplicate records were identified and removed to prevent the same publication from being analyzed more than once. In total, 54 duplicate records were removed. After duplicate removal, the final bibliometric corpus consisted of 181 publications. The inclusion and exclusion criteria are summarized in Table 1.

**Table 1:** Eligibility criteria used in corpus construction.

Category	Criterion	Description
Inclusion	Publication period	Journal articles published between 2001 and 2026 were included.
Inclusion	Language	Only English-language publications were included.
Inclusion	Document type	Only peer-reviewed journal articles were included.
Inclusion	Conceptual and topical relevance	Titles and abstracts were screened to determine whether the records were substantively related to STEM career choice, career aspirations, or vocational interest and were consistent with the broader conceptual scope of the study. Full texts were consulted when relevance could not be determined from titles and abstracts alone.
Inclusion	Search-query eligibility	Records were retrieved when they matched at least one term from each of four conceptual search blocks—STEM-related, psychosocial-construct, gender-related, and career-related terms—within the database-specific searchable fields.
Exclusion	Outside the conceptual or topical scope	Records that were clearly unrelated to STEM career-choice research or fell outside the intended conceptual scope of the study were excluded.
Exclusion	Duplicate record	Records appearing in both Web of Science and Scopus were removed after the eligible datasets were merged.

### 5.4 Data export, integration, and preprocessing

The records retrieved from the WoS and Scopus databases were exported in BibTeX and CSV formats. These formats were selected because they allow the transfer of bibliographic metadata required for bibliometric analysis, including authors, titles, abstracts, keywords, source titles, publication years, citation information, affiliations, and references. Records obtained from the WoS and Scopus databases were consolidated into a single dataset using the bibliometrix package in R and its Biblioshiny web interface. The bibliometrix package, which is widely used for bibliometric analyses, was selected for this process (Aria and Cuccurullo, 2017). Bibliometrix enables the systematic, transparent, and reproducible processing and analysis of bibliographic data. Additionally, the R environment provides a flexible infrastructure for bibliometric studies through its statistical analysis, data management, and visualization capabilities (e.g., Büyükkıdık, 2022; Tamphu et al., 2024). During preprocessing, the records exported from the two databases were converted into a common bibliographic data structure and merged into a single dataset. Database-specific metadata fields were harmonized to ensure that comparable information from WoS and Scopus could be analyzed together. Following the eligibility assessment described in Section 5.3, the 235 retained records from the two databases were integrated into a single dataset. After the datasets were integrated, duplicate records were identified and removed to prevent the same publication from being counted more than once. Duplicate detection was conducted by comparing available bibliographic information such as title, author names, publication year, DOI, and source title. A total of 54 duplicate records were removed, resulting in a final dataset of 181 unique publications. Following duplicate removal, the final dataset was used for the subsequent bibliometric performance and science-mapping analyses. This preprocessing procedure was conducted to ensure that the final corpus was internally consistent, non-redundant, and appropriate for subsequent performance analysis and conceptual structure analysis. All preprocessing and bibliometric analyses were conducted in R version 4.5.2 using the bibliometrix package.

### 5.5 Keyword cleaning and normalization

Before conducting the conceptual structure analyses, a keyword cleaning and normalization procedure was applied to improve the accuracy and interpretability of the keyword-based maps. The analyses were conducted using the cleaned All Keywords field. This field was used as the starting point because it allowed the study to capture both author-provided and database-indexed keyword information available in the imported records. However, because database-generated keyword fields may include non-substantive indexing terms, the keyword set was cleaned before the co-occurrence, thematic map, and thematic evolution analyses were conducted. First, spelling, hyphenation, and singular/plural variants referring to the same concept were standardized through a thesaurus-based procedure. For example, “self-efficacy” was merged under “self-efficacy,” “self-concept” under “self-concept,” “gender-differences” under “gender differences.” Singular and plural variants such as “university” and “universities,” as well as alternative forms such as “math” and “mathematics,” were also harmonized under a single preferred form. This procedure prevented conceptually identical terms from being represented as separate nodes or themes in the keyword-based analyses. Second, non-substantive database or indexing terms that did not represent meaningful conceptual themes in the context of STEM career choice research were removed from the keyword set. These included terms such as “article,” “adult,” “controlled study.” The cleaned and normalized keyword set was subsequently used to rerun the keyword co-occurrence, thematic map, and thematic evolution analyses.

### 5.6 Bibliometric and science-mapping analyses

**Figure 2:**
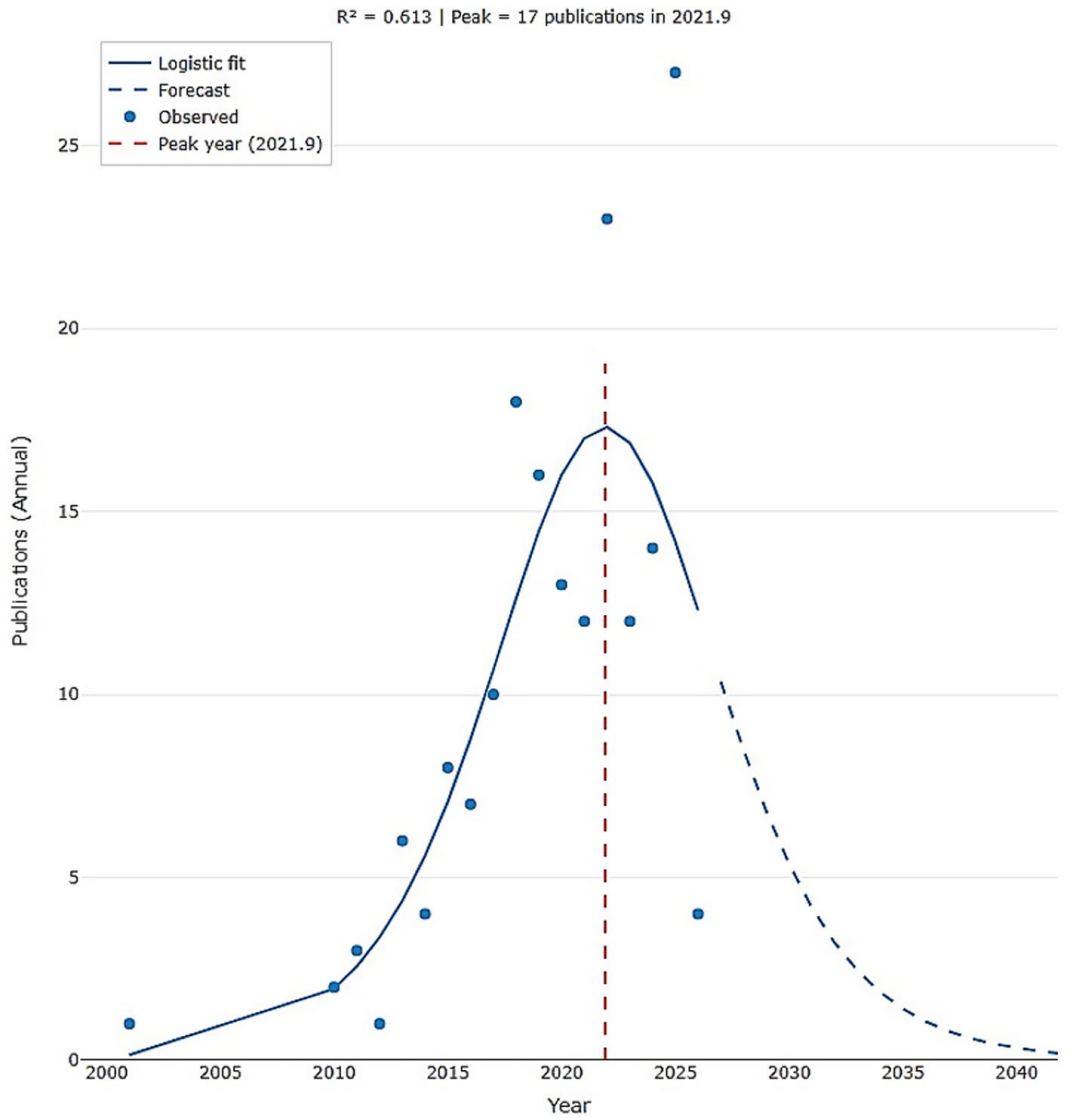
Exploratory life-cycle model of annual scientific production in the analyzed corpus.

The bibliometric and science-mapping analyses were conducted in R using the bibliometrix package and its Biblioshiny interface (Aria and Cuccurullo, 2017). The analyses were organized into two main categories: performance analysis and conceptual structure analysis. This distinction is consistent with established bibliometric frameworks that combine descriptive performance indicators with science-mapping techniques to examine the development and conceptual organization of a research domain (Zupic and Čater, 2015; Donthu et al., 2021). First, performance analysis was conducted to examine the general development and productivity structure of the selected corpus. This stage included annual scientific production, annual growth trends, citation patterns, the most productive authors, source titles, countries, and collaboration patterns. These indicators were used to describe the publication dynamics, productivity distribution, and international structure of the analyzed corpus. Annual scientific production was used to examine changes in publication output over time. The life-cycle analysis presented in Figure 2 was used to visualize the temporal development pattern of annual scientific production in the selected corpus. The life-cycle visualization was generated in Biblioshiny using the “Life Cycle of Scientific Production” module under the Overview menu. The model was based on annual publication counts and was treated as an exploratory visualization of the publication trajectory rather than as a predictive model. Therefore, the fitted curve and its estimated peak were interpreted cautiously and distinguished from the observed annual publication counts. The model fit was moderate (*R*^2^ = 0.613), indicating that the curve should not be interpreted as strong evidence of field maturation or as a reliable long-term projection. Second, conceptual structure analyses were conducted to examine the keyword-based thematic organization and conceptual development of the selected corpus. These analyses included keyword co-occurrence analysis, thematic mapping, and thematic evolution analysis. The keyword co-occurrence analysis was used to identify clusters of frequently connected concepts within the selected keyword network. The thematic map was used to position themes according to centrality and density, following the conventional interpretation of motor themes, niche themes, basic/transversal themes, and emerging or declining themes. The thematic evolution analysis was used to examine how the main themes changed across the two periods defined in the study, 2001–2013 and 2014–2026. All conceptual structure analyses were conducted using the cleaned keyword set described in Section 5.5. This ensured that spelling variants, hyphenation differences, singular/plural forms, and non-substantive indexing terms did not distort the keyword-based maps. The results of these analyses were visualized and interpreted to identify the main conceptual clusters, thematic positions, and temporal transformations within the selected corpus. Because these analyses were based on keyword co-occurrence and thematic relationships, the resulting maps were interpreted as representations of the conceptual structure of the selected corpus rather than as evidence that the focal concepts were equally central empirical variables in every publication.

### 5.7 Dataset characteristics

Basic information regarding the final dataset is presented in Table 2. As summarized in Table 2, the final corpus comprised 181 publications published between 2001 and 2026 across 108 sources and involved 472 authors. The annual growth rate was 5.7%, the average document age was 5.9 years, and the average number of co-authors per document was 2.81. International co-authorship represented 13.26% of the corpus. The mean number of citations per document was 49.28. Because citation distributions may be influenced by a relatively small number of highly cited publications, this mean value should be interpreted cautiously as an overall average rather than as the typical citation count of a publication in the corpus.

**Table 2:** Main information about the data collection.

Description	Results
Total number of documents	181
Time period	2001–2026
Number of sources (journals, etc.)	108
Number of authors	472
Average citations per document	49.28
Annual growth rate (%)	5.7
Average document age	5.9
Average number of co-authors per document	2.81
International collaboration (%)	13.26

## 6 Results

### 6.1 Descriptive and performance analysis of the bibliometric corpus

In this section, the descriptive characteristics of the bibliometric corpus were examined within the scope of bibliometric analysis. Accordingly, trends in scientific output over the years, the publication trajectory of the corpus, the most productive authors and sources, and the distribution of scientific output by country were analyzed. Additionally, by examining the distribution of corresponding authors by country, the geographical structure of research output and the patterns of international co-authorship were described. These analyses characterize the temporal development, leading contributors, publication outlets, and geographical distribution of the corpus constructed through the predefined search and screening criteria.

**Figure 3:**
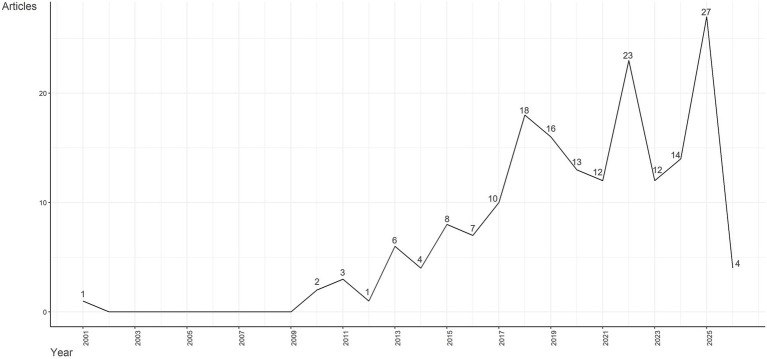
Annual scientific production in the analyzed corpus.

Figure 3 presents the annual distribution of scientific production in the analyzed corpus.

When the number of publications by year is examined in Figure 3, it can be seen that publication activity was very limited during the early years and reached higher, although fluctuating, levels after 2017. One publication was recorded in 2001, no publications were recorded between 2002 and 2009, and scientific output resumed in 2010. The highest number of publications was reached in 2025, with 27 publications. The low figure for 2026 reflects the fact that 2026 was an incomplete publication year at the time of data collection and should not be interpreted as evidence of a definitive decline in publication activity. These findings indicate that annual scientific production generally increased within the selected corpus over the study period, although the pattern was characterized by year-to-year fluctuations.

Figure 2 presents the exploratory life-cycle model of annual scientific production in the analyzed corpus.

Figure 2 presents an exploratory life-cycle model of annual scientific production. The fitted logistic curve indicated an estimated model peak of approximately 17 publications per year around 2021.9, with a moderate model fit (*R*^2^ = 0.613). This modeled peak should be distinguished from the observed annual publication peak of 27 publications in 2025, as shown in Figure 3. Because the dataset includes 2026, which was an incomplete publication year at the time of data collection, and because the model fit was moderate, the modeled trajectory was interpreted cautiously. Accordingly, the figure was used as an exploratory visualization of the publication trajectory of the selected corpus rather than as a definitive representation of future publication patterns.

**Figure 4:**
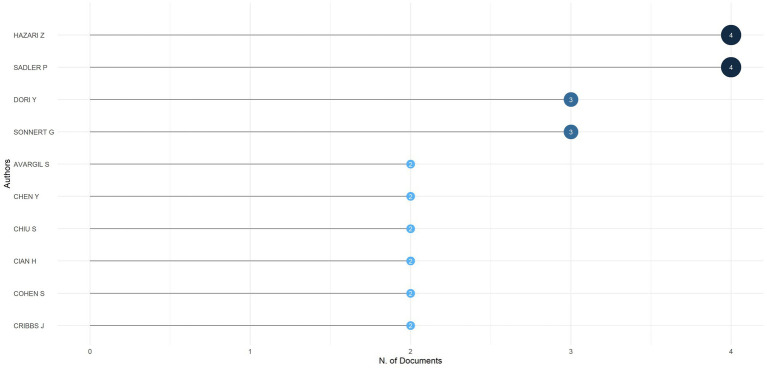
Most productive authors in the analyzed corpus.

The most productive authors in the analyzed corpus are shown in Figure 4.

Figure 4 presents the 10 most productive authors in the analyzed corpus. Hazari and Sadler jointly ranked first, with four publications each, followed by Dori and Sonnert, with three publications each. Avargil, Chen, Chiu, Cian, Cohen, and Cribbs each contributed two publications. The relatively narrow range of publication counts among the leading authors indicates that author productivity within the corpus was not strongly concentrated around a single researcher.

**Figure 5:**
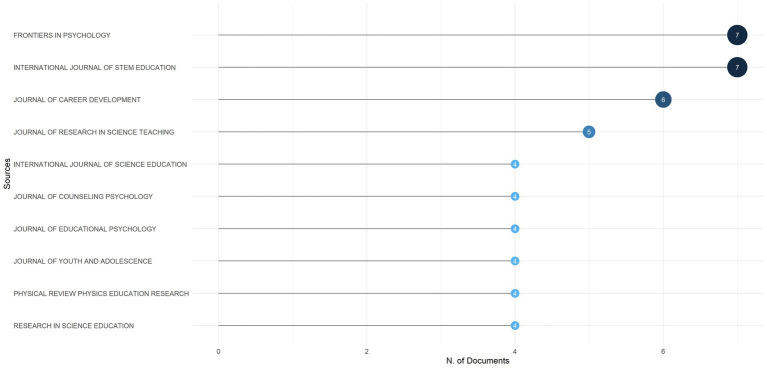
Most relevant sources in the analyzed corpus.

The most relevant sources in the analyzed corpus are presented in Figure 5.

Figure 5 shows that “Frontiers in Psychology” and “International Journal of STEM Education” jointly ranked first, with seven publications each. These journals were followed by the “Journal of Career Development,” with six publications, and the “Journal of Research in Science Teaching,” with five publications. The remaining six sources in the top 10 each contributed four publications. Although several leading outlets can be identified, the corpus was distributed across 108 different sources, indicating that publication activity was spread across a broad range of journals. The disciplinary scope of the leading sources, which includes psychology, STEM and science education, and career development, suggests that the publications included in the selected corpus are distributed across several related research domains.

**Figure 6:**
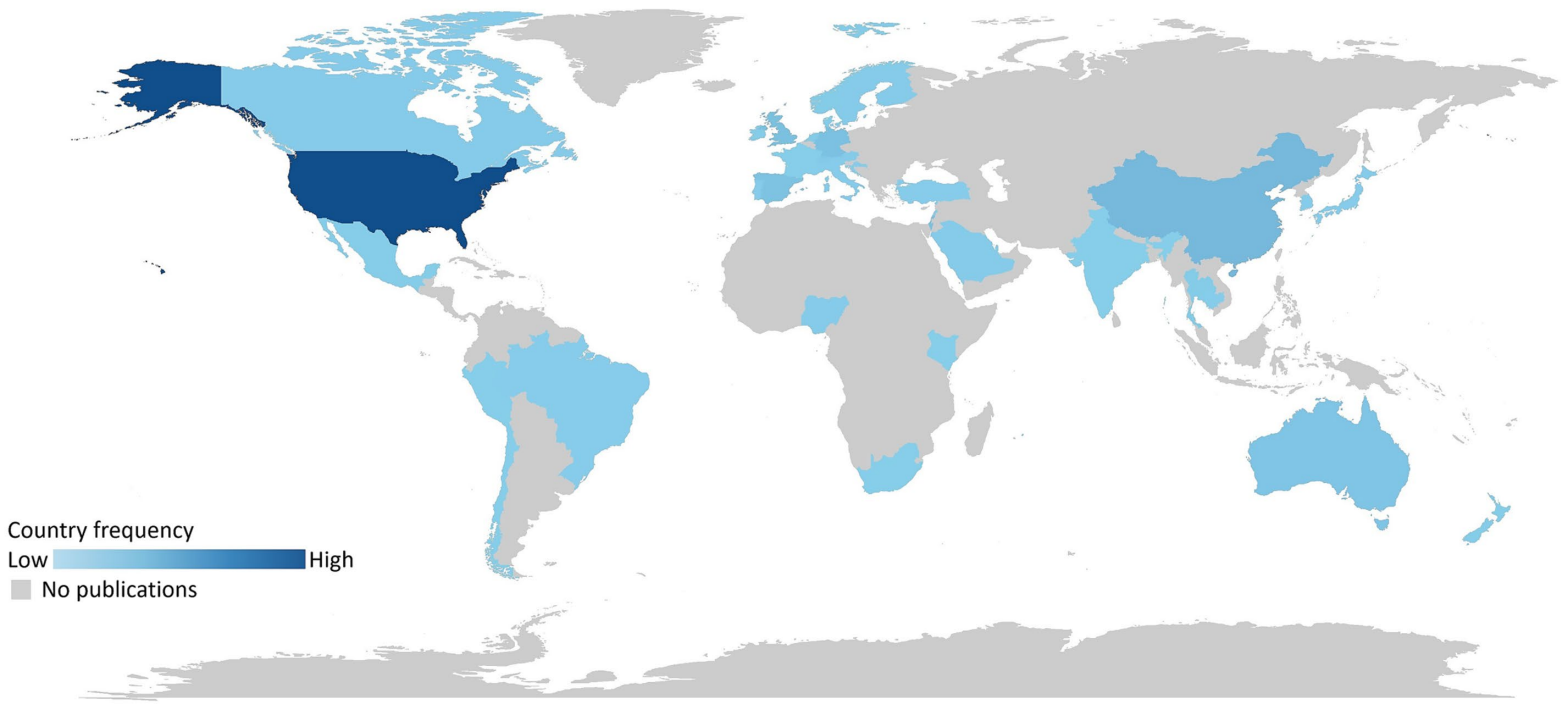
Geographical distribution of country occurrences based on authors’ affiliation information in the analyzed corpus.

The geographical distribution of country occurrences based on authors’ affiliation information is shown in Figure 6.

Figure 6 presents the geographical distribution of country occurrences based on the frequency of country occurrences derived from the authors’ affiliation information. The United States had the highest country frequency (*n* = 248), followed by China (*n* = 39) and the United Kingdom (*n* = 25). Germany and Israel each had a frequency of 24, followed by Spain (*n* = 20) and Australia (*n* = 17). Together, these seven countries accounted for 397 of the 489 country occurrences (81.2%), indicating that the geographical representation of the corpus was concentrated in a relatively small group of countries. These frequencies reflect country occurrences in the affiliation metadata and should not be interpreted as counts of unique publications.

**Figure 7:**
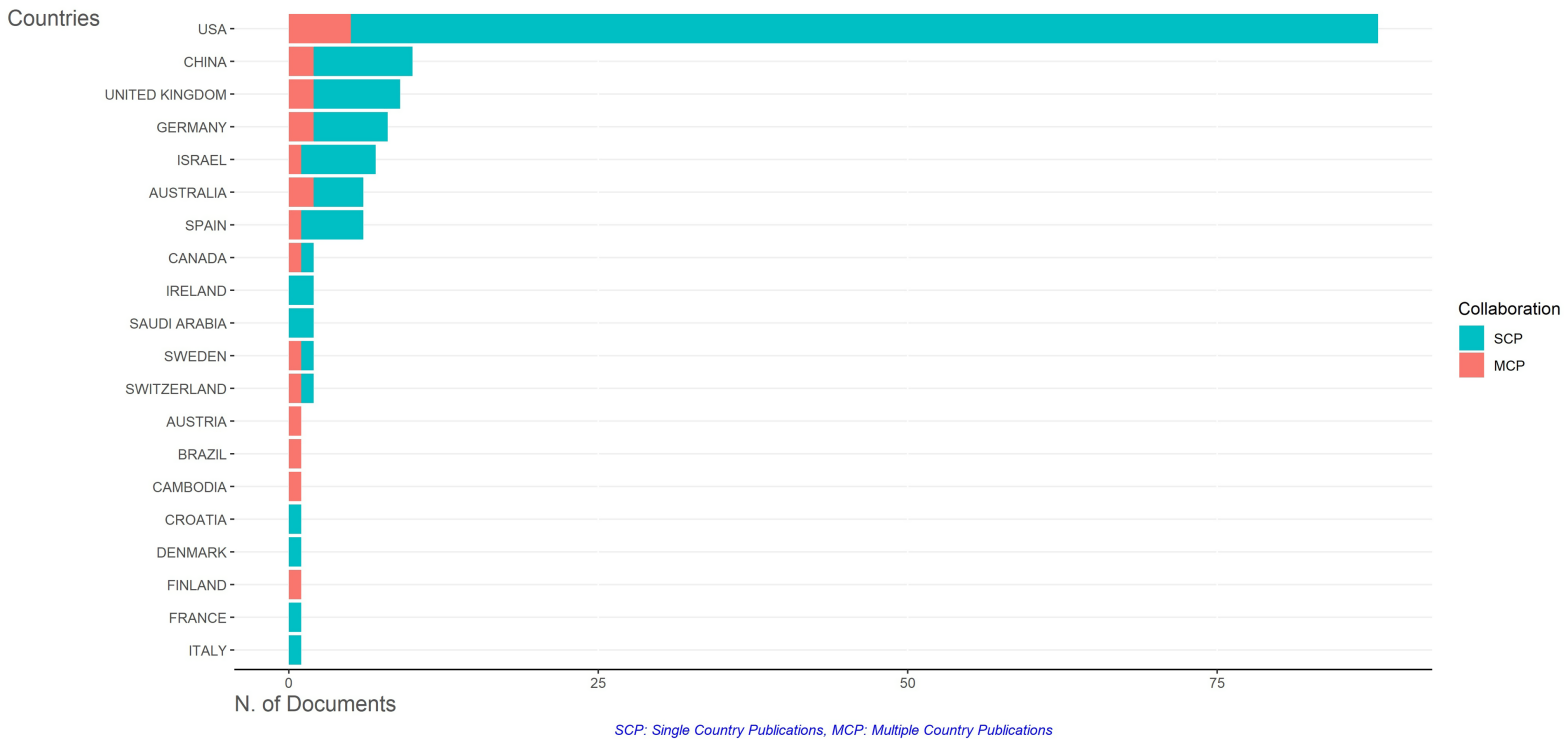
Publication and collaboration patterns by corresponding author’s country in the analyzed corpus.

Figure 7 shows publication and collaboration patterns by corresponding author’s country.

Figure 7 presents the distribution of single-country publications (SCP) and multiple-country publications (MCP) according to the corresponding author’s country. The United States had the highest number of publications, with 88 publications comprising 83 SCPs and 5 MCPs. It was followed by China with 10 publications (8 SCPs and 2 MCPs), the United Kingdom with 9 publications (7 SCPs and 2 MCPs), and Germany with 8 publications (6 SCPs and 2 MCPs). Israel contributed 7 publications, including 6 SCPs and 1 MCP. Australia and Spain each contributed 6 publications; Australia had 4 SCPs and 2 MCPs, whereas Spain had 5 SCPs and 1 MCP. Canada, Ireland, Saudi Arabia, Sweden, and Switzerland each contributed two publications. Canada, Sweden, and Switzerland each had one SCP and one MCP, whereas both publications from Ireland and Saudi Arabia were SCPs. Austria, Brazil, Cambodia, Croatia, Denmark, Finland, France, and Italy each contributed one publication. The publications associated with Austria, Brazil, Cambodia, and Finland were MCPs, whereas those associated with Croatia, Denmark, France, and Italy were SCPs. Overall, the distribution shown in Figure 7 indicates that SCPs constituted the larger share of the publication output among the leading corresponding-author countries represented in the selected corpus.

### 6.2 Conceptual structure of the analyzed corpus

In this section, the conceptual structure of the analyzed corpus was examined using the cleaned keyword set. Following the keyword cleaning and normalization procedure described in the Methods section, keyword co-occurrence analysis, thematic mapping, and thematic evolution analysis were conducted to identify the conceptual patterns, thematic clusters, and temporal patterns of organization within the corpus. These analyses provide insights into how the selected literature is organized around STEM career choice and how gender, identity, self-efficacy, and self-concept are represented within its keyword-based conceptual structure. Because the corpus was constructed through queries combining STEM-related, psychosocial-construct, gender-related, and career-related search blocks, the prominence of these concepts in the conceptual maps should be interpreted in relation to the study’s search design. Their visibility in the maps does not necessarily indicate that they were equally central empirical variables in every publication.

#### 6.2.1 Keyword co-occurrence network

**Figure 8:**
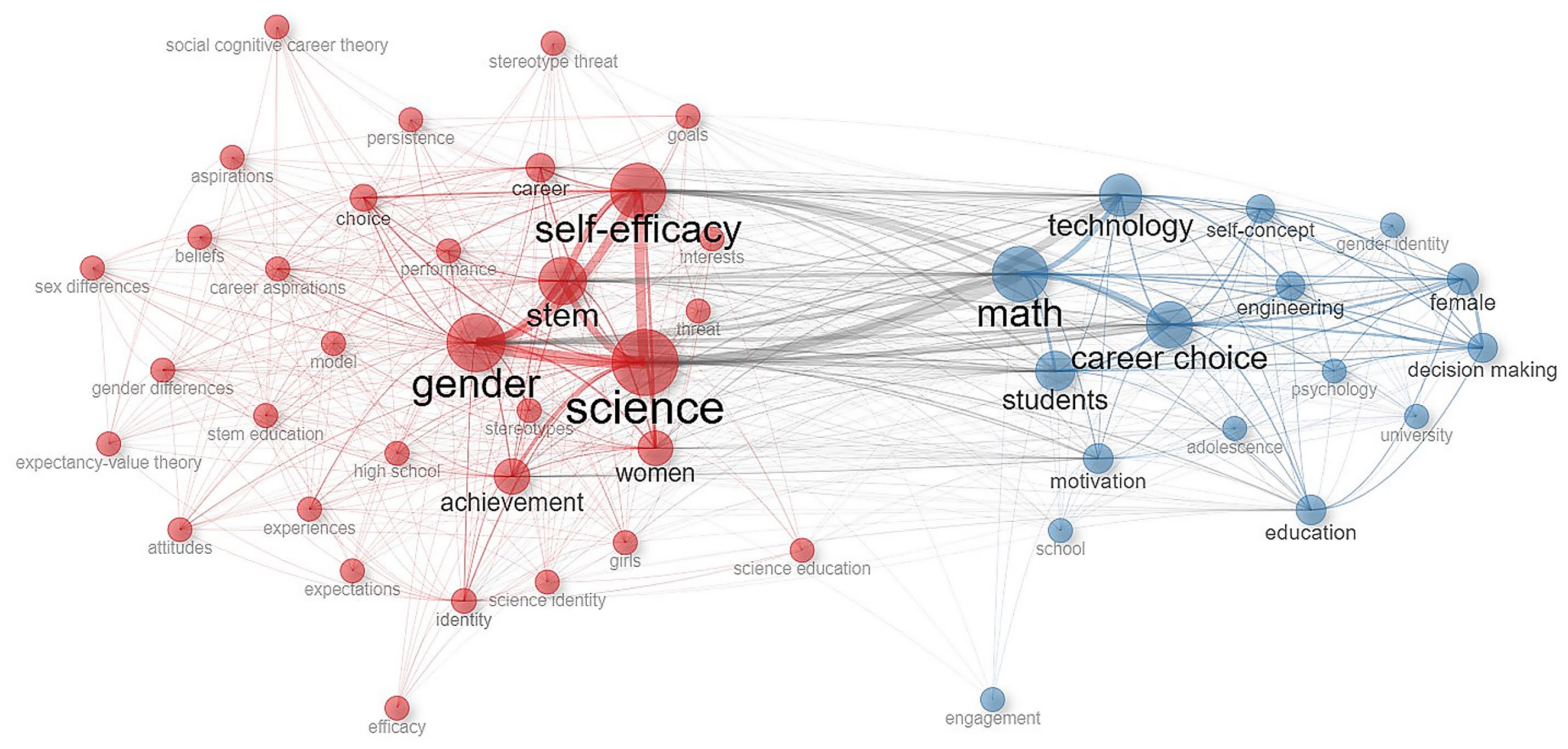
Keyword co-occurrence network in the analyzed corpus.

The keyword co-occurrence network is shown in Figure 8.

When the keyword co-occurrence network in Figure 8 is examined, it is observed that the cleaned keyword set is organized around two main conceptual clusters. The first cluster, shown in red, is centered on concepts such as “science,” “gender,” “STEM,” “self-efficacy,” “women,” “achievement,” “identity,” “science identity,” “stereotypes,” and “career aspirations.” This cluster brings together terms associated with psychosocial and educational dimensions of STEM career research, including gender-related participation, efficacy beliefs, identity-related constructs, achievement, and career aspirations. The second cluster, shown in blue, is structured around concepts such as “career choice,” “students,” “math,” “technology,” “decision making,” “education,” “engineering,” “self-concept,” “psychology,” “female,” “gender identity,” and “university.” This cluster brings together career-choice terms with disciplinary, educational, and student-related contexts. The representation of self-concept within this cluster indicates that it co-occurs with career- and discipline-oriented terms in the selected keyword network.

The connections between the two clusters indicate that the terms associated with psychosocial processes and career-oriented research strands do not occur in completely separate parts of the network. Rather, the network shows keyword-based connections among gender, self-efficacy, self-concept, identity, and STEM career choice within the selected corpus. However, because the corpus was constructed using mandatory STEM-related, psychosocial-construct, gender-related, and career-related search blocks, the prominence of these concepts in the network should be interpreted in light of the study’s search design. Therefore, the co-occurrence network should be understood as mapping the conceptual organization of the selected corpus rather than as evidence of the overall centrality of these constructs across the entire STEM career literature or of causal relationships among them.

#### 6.2.2 Thematic map

**Figure 9:**
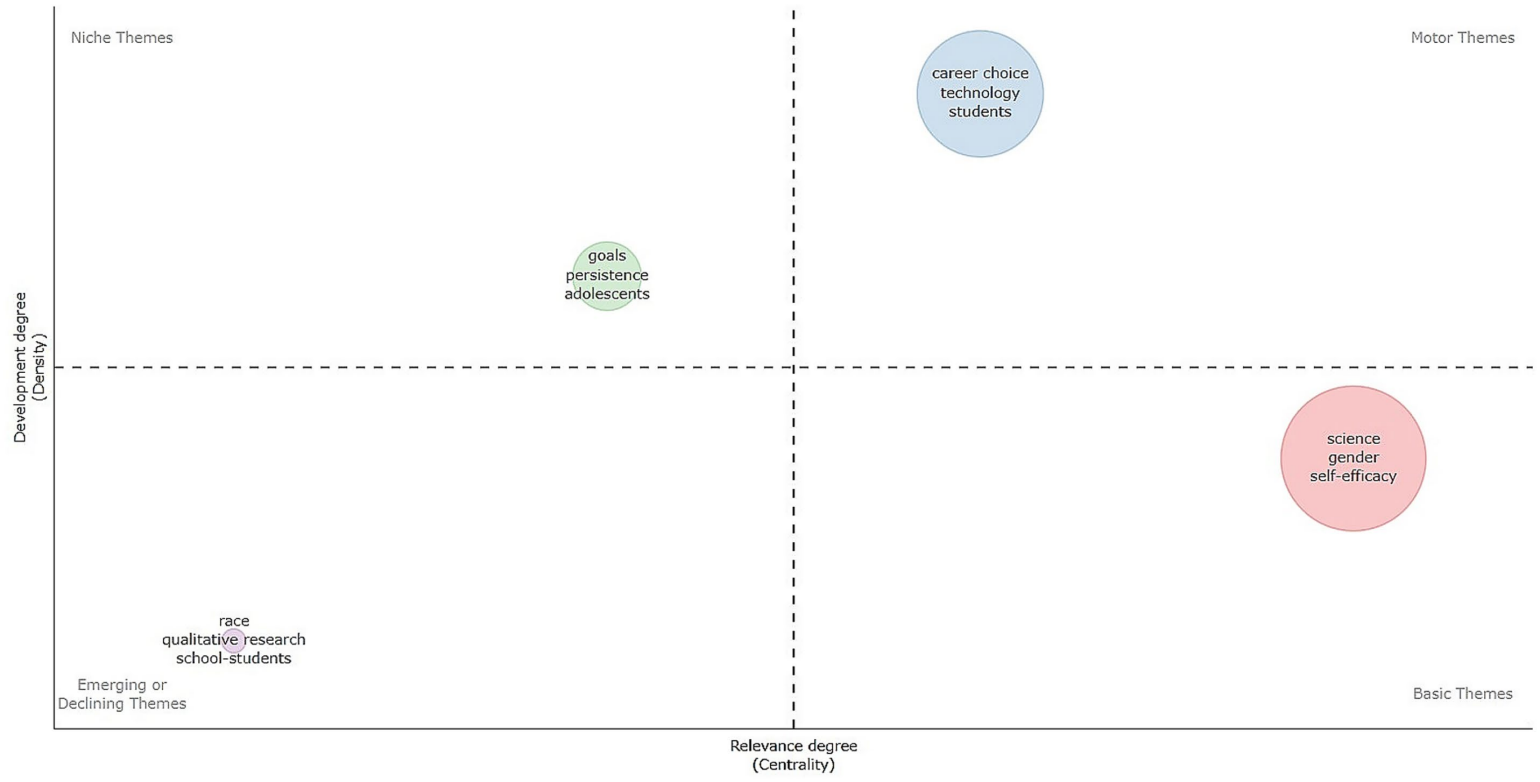
Thematic map of the analyzed corpus.

The thematic map of the analyzed corpus is presented in Figure 9.

Figure 9 positions the themes generated from the cleaned and normalized keyword set according to their centrality and density. Following the conventional interpretation of thematic maps (Cobo et al., 2012), the upper-right quadrant contains motor themes, the upper-left quadrant contains niche themes, the lower-right quadrant contains basic or transversal themes, and the lower-left quadrant contains emerging or declining themes.

The cluster represented by “career choice,” “technology,” and “students” is located in the motor-themes quadrant. Its position indicates that this theme is both highly connected to the overall conceptual structure and comparatively well developed internally. Career choice therefore appears as a central and developed thematic area within the selected corpus, closely associated with students and technology-related educational and disciplinary contexts. The cluster represented by “science,” “gender,” and “self-efficacy” is positioned in the basic or transversal themes quadrant. This location indicates high centrality but comparatively lower internal density. These concepts can therefore be interpreted as broad conceptual foundations that connect with several areas of the analyzed literature, although they do not form an equally consolidated and internally specialized thematic structure. The cluster represented by “goals,” “persistence,” and “adolescents” appears in the niche-themes quadrant. This position suggests that the theme is internally developed but less strongly connected with the overall conceptual structure of the corpus. It therefore represents a more specialized research strand concerned with goal formation, persistence, and adolescent participation in STEM-related educational and career pathways. A fourth cluster, represented by “race,” “qualitative research,” and “school students,” is located in the emerging or declining themes quadrant. This position indicates relatively low centrality and density. However, the thematic map alone does not determine whether the cluster is newly emerging or losing prominence. It should therefore be interpreted cautiously as a peripheral and comparatively underdeveloped thematic area that may reflect growing attention to race, qualitative approaches, and school-level student experiences.

Overall, the thematic map shows that the analyzed corpus is organized around a developed motor theme connecting career choice, technology, and students; a broad basic theme linking science, gender, and self-efficacy; a specialized niche theme concerning goals, persistence, and adolescents; and a peripheral emerging or declining theme involving race, qualitative research, and school students. The resulting structure indicates that disciplinary and career-related orientations coexist with broad psychosocial and educational themes, together with more specialized lines of inquiry within the selected corpus. These thematic positions describe the internal organization of the keyword network and should not be interpreted as direct measures of the empirical importance of the associated constructs.

#### 6.2.3 Thematic evolution

**Figure 10:**
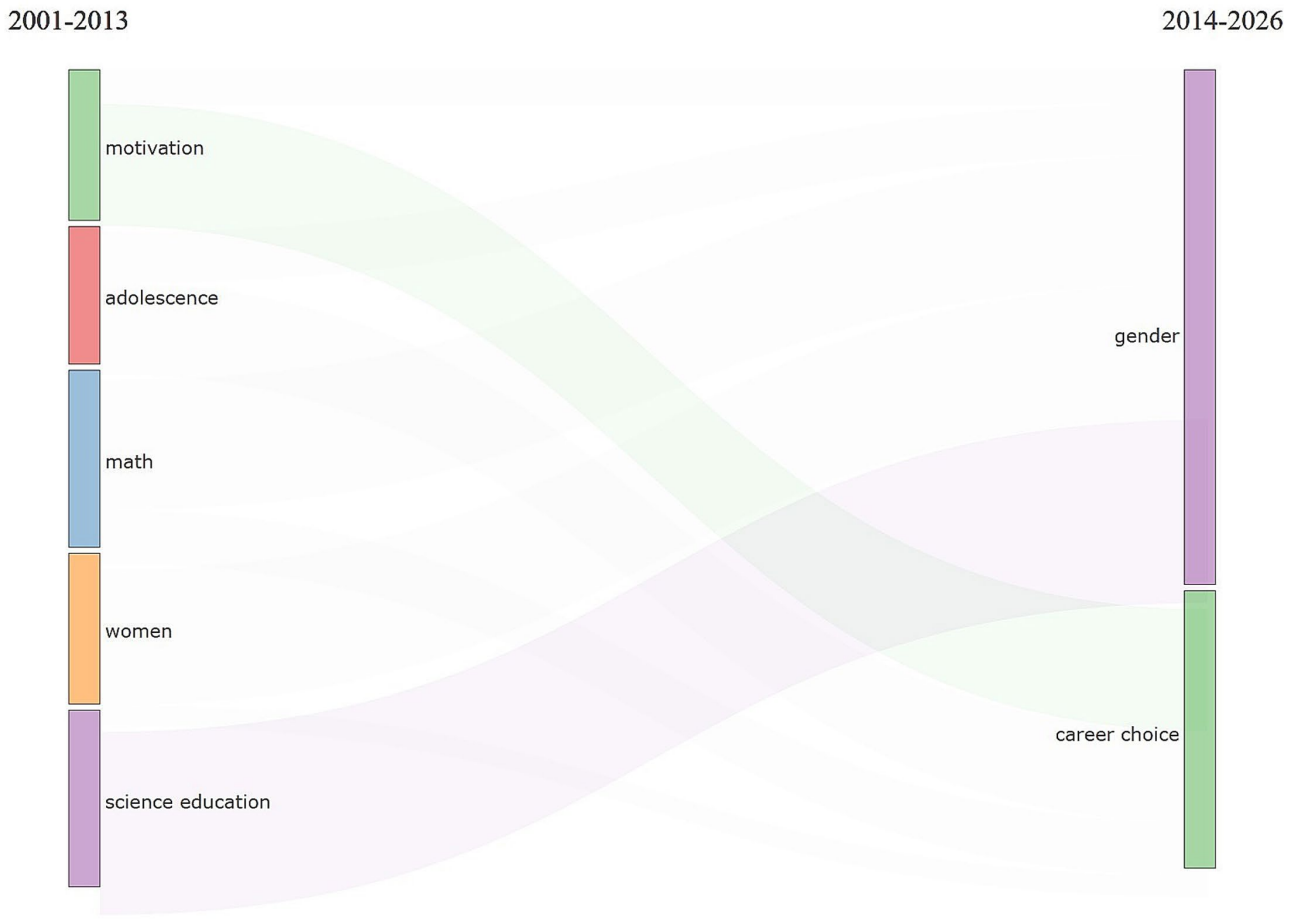
Thematic evolution of the analyzed corpus.

The thematic evolution of the analyzed corpus is illustrated in Figure 10.

Figure 10 presents the thematic evolution analysis based on the cleaned and normalized keyword set and illustrates how the thematic structure of the selected corpus was reorganized between the 2001–2013 and 2014–2026 periods. In the first period, the thematic structure was represented by “motivation,” “adolescence,” “math,” “women,” and “science education.” These themes suggest that earlier studies in the corpus were organized around motivational processes, developmental stages, disciplinary contexts, women’s participation, and science-education settings. In the second period, the thematic structure appears more concentrated around “gender” and “career choice.”

The connection between “women” in the first period and “gender” in the second period indicates thematic continuity accompanied by a broader reorganization of gender-related terminology; it should not be interpreted as the complete replacement of a population-specific focus by a broader analytical category. Similarly, the links connecting several earlier themes to “career choice” suggest that motivational, developmental, and disciplinary concerns became increasingly associated with career-related decision making in the later period. These links are based on shared keywords across periods and represent thematic continuity and reorganization rather than causal or developmental pathways among the concepts.

Overall, the thematic evolution indicates a shift from a relatively dispersed structure involving motivation, adolescence, math, women, and science education toward a later structure more explicitly organized around gender and career choice. This pattern suggests that gender-related perspectives and career decision making became more visible organizing themes within the selected literature. However, because the corpus was constructed using mandatory gender- and career-related search terms, these thematic developments should be interpreted in relation to the study’s search design. They describe changes in the organization and visibility of themes within the selected corpus rather than the evolution of the entire STEM career-choice literature. In addition, the second period includes 2026, which was incomplete at the time of data collection; therefore, the most recent thematic patterns should be interpreted cautiously.

## 7 Discussion

This study mapped the bibliometric, geographical, and conceptual development of a deliberately delimited corpus of STEM career-choice research constructed through predefined search and screening criteria. The findings indicate that annual publication output generally increased over the study period, although this development was characterized by substantial year-to-year fluctuations. Publication activity was very limited during the early years, resumed in 2010, and reached consistently higher levels after 2017. The highest observed annual output occurred in 2025, with 27 publications. The lower number recorded for 2026 should not be interpreted as evidence of a decline because data collection was completed before the publication year had ended. The overall increase is consistent with the growing scholarly attention given to psychosocial and contextual dimensions of STEM career development, including self-efficacy, self-concept, identity, and gender-related experiences (Luo et al., 2021; Wang et al., 2023; Chen et al., 2024; Lambert et al., 2024; Küçükaydın and Ulum, 2025).

The exploratory life-cycle analysis provides a complementary, but more cautious, perspective on the publication trajectory. The fitted model estimated a peak of approximately 17 publications around 2021.9, whereas the actual annual data showed an observed peak of 27 publications in 2025. This distinction is important because the moderate model fit (*R*^2^ = 0.613) and the inclusion of an incomplete publication year limit the model’s capacity to represent the future development of the literature. Accordingly, the model should not be interpreted as demonstrating that the corpus has already entered a period of decline or disciplinary maturation. Rather, it offers an exploratory representation of the publication trajectory, while the observed data indicate that research activity remained substantial in the most recent complete years.

The distribution of author productivity suggests that the analyzed literature has developed through the contributions of a relatively broad research community rather than around one clearly dominant scholar. Hazari and Sadler were the most productive authors, with four publications each, but the differences among the leading authors were relatively small. This pattern is consistent with the multidimensional nature of the topic, which has been examined across different educational levels, national contexts, STEM disciplines, and psychosocial perspectives (e.g., Avargil et al., 2020; Chen et al., 2024; Lambert et al., 2024; Tal et al., 2024). The distribution across 108 publication sources further demonstrates that the corpus is not confined to a single disciplinary outlet. The presence of journals specializing in psychology, career development, STEM education, science education, and adolescence indicates that STEM career choice is studied across several related research domains (e.g., Li et al., 2021; Chen et al., 2024; Yamani and Almazroa, 2024). This interdisciplinary distribution is particularly relevant from an educational psychology perspective because the literature combines questions of learning, motivation, self-beliefs, identity development, educational participation, and career decision-making.

The geographical findings indicate that the representation of the corpus was concentrated in a relatively small group of countries. Based on author-affiliation country occurrences, the United States had the highest frequency, followed by China, the United Kingdom, Germany, Israel, Spain, and Australia. Together, these countries accounted for a substantial majority of all country occurrences. This uneven distribution is broadly consistent with wider observations concerning geographical disparities in scientific production and the concentration of research capacity in particular national systems (UNESCO, 2021; Marginson, 2022). However, the observed concentration may also partly reflect the English-language restriction and the coverage and indexing practices of WoS and Scopus, which may underrepresent scholarship published in other languages or indexed in regional databases. The corresponding-author analysis also showed that single-country publications constituted a larger share of output than multiple-country publications among the leading countries. This finding should not be interpreted as demonstrating that international collaboration is necessarily inadequate, because no disciplinary or cross-field benchmark was applied. It does, however, indicate that nationally based authorship structures were more common than multinational authorship structures within the analyzed corpus.

The keyword co-occurrence network revealed two connected conceptual clusters. The first cluster was organized around science, gender, STEM, self-efficacy, women, achievement, identity, science identity, stereotypes, and career aspirations. This cluster reflects a psychosocial and educational research strand in which STEM career development is considered in connection with gendered experiences, self-beliefs, identity formation, achievement, and motivational processes. This interpretation is consistent with studies linking STEM-related aspirations and choices with self-efficacy, identity, recognition, and gendered educational experiences (Hazari et al., 2010; Wang and Degol, 2017; Wegemer and Eccles, 2019; Jiang et al., 2024; Myint and Robnett, 2024; Küçükaydın and Ulum, 2025). The second cluster was organized around career choice, students, math, technology, decision making, education, engineering, self-concept, psychology, gender identity, and university. This cluster represents a more career- and discipline-oriented strand of the literature. The positioning of self-concept within this cluster shows that the term co-occurs with career decision-making and disciplinary contexts, particularly those involving math, technology, and engineering. However, self-concept did not emerge as an independent central theme in the thematic map or thematic evolution analysis. It should therefore be understood as a relevant but comparatively less visible component of the conceptual structure rather than as a construct occupying the same thematic position as gender or self-efficacy. More broadly, the connections between the two clusters indicate that psychosocial constructs and career-oriented concepts do not occupy completely separate areas of the keyword network. Instead, the network represents self-related beliefs and identity-related terms in connection with educational and occupational orientations. Because gender-related and psychosocial terms were required components of the search strategy, their prominence in the network partly reflects the design and conceptual boundaries of the selected corpus and should not be generalized to the entire STEM career literature. Their visibility also does not necessarily mean that they were examined as primary or equally central empirical variables in every publication.

For the broader STEM career literature, the two-cluster structure suggests that psychosocial explanations and career- or discipline-oriented research have developed as connected but partly differentiated strands. This configuration is consistent with research showing that STEM career development is shaped not only by achievement and disciplinary participation but also by efficacy beliefs, identity, recognition, perceived competence, and gendered educational experiences (Hazari et al., 2010; Wang and Degol, 2017; Wegemer and Eccles, 2019; Jiang et al., 2024; Lu et al., 2024). The different positioning of self-efficacy and self-concept is also theoretically relevant because self-efficacy concerns perceived capability to perform specific tasks, whereas self-concept involves broader evaluations of competence within a domain (Bong and Skaalvik, 2003; Marsh, 2007). Their positions therefore highlight the importance of preserving this distinction when examining STEM aspirations and career decisions.

The thematic map provides further insight into the relative positions of these research strands. The cluster represented by career choice, technology, and students was positioned as a motor theme, indicating relatively high centrality and internal development within the corpus. This position suggests that research connecting students with technology-related contexts and career choice constitutes a comparatively developed and well-connected thematic area. Career choice therefore appears as an organizing theme linked to students’ educational and disciplinary contexts within the corpus. By contrast, the cluster represented by science, gender, and self-efficacy appeared as a basic or transversal theme. Its position indicates that these concepts are broadly connected with other areas of the corpus but are less internally developed than the motor theme. Gender and self-efficacy may therefore be understood as broad transversal concepts within the selected corpus rather than as a narrowly specialized and internally homogeneous theme. This position is compatible with existing research showing that self-efficacy and gender-related experiences are associated with STEM interests, aspirations, choices, and persistence across different educational and disciplinary settings (Lent et al., 1994; Wang and Degol, 2017; Luo et al., 2021). The cluster represented by goals, persistence, and adolescents was located in the niche-themes quadrant. Its relatively high internal development but lower connection to the wider conceptual structure suggests a more specialized research strand concerned with motivational continuity and adolescent participation in STEM pathways. Goals and persistence are also relevant to SCCT, which links self-efficacy, outcome expectations, interests, and goals with career choice and sustained participation (Lent et al., 1994; Lent and Brown, 2019). However, the niche position of this cluster indicates that these concepts form a more bounded line of inquiry within the selected corpus rather than a broadly transversal theme. A further cluster represented by race, qualitative research, and school students was positioned in the emerging or declining themes quadrant. This location indicates comparatively low centrality and internal development. The thematic map alone cannot determine whether this cluster is newly emerging or losing prominence. It is therefore more appropriate to interpret it cautiously as a peripheral methodological and contextual research strand connecting race-related perspectives, qualitative approaches, and school-level student experiences. Its presence nevertheless highlights an area that may warrant further investigation, particularly because qualitative research can provide detailed evidence about how students interpret belonging, recognition, stereotypes, and identity within specific social and educational contexts. From an SCCT perspective, the thematic structure indicates that several SCCT-related constructs and processes are represented across distinct thematic strands rather than within a single integrated configuration. The basic or transversal position of the science–gender–self-efficacy theme is consistent with the broad relevance of self-efficacy within SCCT’s interest and choice models, whereas the niche position of the goals–persistence–adolescents theme indicates that goals and persistence constitute a more specialized and less broadly connected line of inquiry within the analyzed corpus (Lent et al., 1994; Lent and Brown, 2019). Identity, self-concept, and gender-related experiences add complementary interpretive dimensions by foregrounding recognition and identification, broader competence evaluations, and sociocultural and contextual influences on STEM participation (Carlone and Johnson, 2007; Parker et al., 2014; Wang and Degol, 2017; Dou et al., 2019). These findings do not test the directional or causal relationships proposed by SCCT; rather, they show how SCCT-related and complementary psychosocial constructs are represented and thematically connected within the selected literature.

The thematic evolution findings suggest a consolidation and reframing of the selected literature rather than a simple replacement of earlier research interests. The continuity between “women” and “gender” may reflect a broadening from population-specific questions concerning women’s participation toward the use of gender as a wider analytical lens. At the same time, the links from earlier themes related to motivation, adolescence, mathematics, women, and science education to the later themes of gender and career choice suggest that previously more dispersed developmental, motivational, disciplinary, and demographic concerns became more concentrated around gendered participation and career decision-making. From an educational psychology perspective, this reorganization points to a more focused framing of STEM career development in which gendered experiences, motivational processes, developmental contexts, and disciplinary settings are considered in relation to students’ career orientations. Although these findings are based on the selected corpus rather than the entire STEM career literature, they are relevant to broader discussions in STEM career research because they suggest that STEM participation is increasingly framed not only as a matter of disciplinary interest or educational exposure, but also as a gendered career-development and decision-making issue. However, this apparent consolidation should not be interpreted as a field-wide developmental trajectory. Because gender- and career-related terminology were mandatory components of the search strategy, their later prominence partly reflects the conceptual boundaries of the selected corpus.

Taken together, the findings indicate that disciplinary, career-related, psychosocial, and contextual strands are connected within the selected literature. More broadly, this pattern suggests that STEM career research is moving toward a more integrated account of career development. From an educational psychology perspective, this structure highlights the value of examining STEM career development not only through achievement and disciplinary participation but also through capability beliefs, competence perceptions, goals, identities, recognition, and gendered educational experiences. The findings also indicate that some methodological and contextual themes remain comparatively peripheral within the selected corpus. These findings describe the conceptual organization of the deliberately delimited corpus; they do not establish causal effects or validate a particular theoretical model. They should also be interpreted in light of the four-block search strategy, which provided a focused corpus but may have excluded relevant STEM career studies using adjacent terminology or different keyword formulations. Nevertheless, they provide a basis for more focused, theory-informed empirical investigation of the relationships represented in the mapped literature.

## 8 Conclusion

This study examined the bibliometric and conceptual structure of a deliberately delimited corpus of STEM career-choice research constructed through predefined search and screening criteria. By combining records from WoS and Scopus and integrating performance analysis with science-mapping techniques, the study mapped the publication development, geographical distribution, collaboration patterns, conceptual organization, and thematic evolution of the selected literature. Annual scientific output generally increased over the study period, particularly after 2010, although publication activity fluctuated across years. The lower output recorded for 2026 should be interpreted cautiously because data collection was completed before the end of that publication year.

The conceptual findings indicate that the keyword-based structure of the selected literature is organized around two interconnected strands: one emphasizing psychosocial and educational processes and another emphasizing career choice, disciplinary orientations, and educational contexts. The thematic map further shows that career choice, technology, and students constitute a comparatively developed motor theme, whereas science, gender, and self-efficacy function as broad basic or transversal themes. More specialized and peripheral strands concern goals and persistence among adolescents and a cluster involving race, qualitative research, and school-level student experiences. Self-concept is represented in connection with career- and discipline-oriented research but does not emerge as an equally prominent independent thematic anchor. The thematic evolution analysis suggests that the earlier literature was distributed across motivation, adolescence, math, women, and science education, whereas the later period was more explicitly organized around gender and career choice. This development reflects thematic continuity and reorganization within the selected corpus rather than the complete replacement of earlier research interests. It also indicates that gender-related perspectives and career decision making became more visible organizing themes in the later period. Because gender-related, career-related, and psychosocial terms were incorporated into the search design, these patterns should be interpreted as characteristics of the selected corpus rather than as unrestricted representations of the entire STEM career-choice literature.

## 9 Limitations and future research

The findings should be interpreted in light of several limitations. First, the corpus was limited to English-language journal articles indexed in WoS and Scopus. Although combining these databases provided broader coverage than using a single source, their indexing policies may have affected the geographical distribution, publication-source patterns, and collaboration results. This may have introduced coverage-related limitations, because studies published in journals not indexed by WoS or Scopus, or indexed differently across the two databases, may have been underrepresented or absent from the corpus. Research published in other languages, regional databases, conference proceedings, books, and book chapters was not included. In addition, differences between the searchable fields and indexing practices of WoS and Scopus may have influenced record retrieval, even though equivalent conceptual search blocks were applied in both databases. Future studies could incorporate additional disciplinary and regional databases to examine whether different geographical and conceptual patterns emerge.

Second, the corpus was deliberately constructed by combining four conceptual search blocks: STEM-related terms, career-related terms, gender-related terms, and psychosocial-construct terms. Because the four blocks were connected using the AND operator, each retrieved record was required to match at least one term from each block within the database-specific searchable fields. The psychosocial block included identity, self-efficacy, and self-concept as alternative terms. However, matching these search blocks did not necessarily mean that all focal concepts were examined as primary or equally central empirical variables in every publication. In some records, particular terms may have appeared as contextual, secondary, or database-indexed information rather than as the principal focus of the study. Consequently, the study does not represent all factors associated with STEM career choice, and the visibility of the focal constructs partly reflects the search design. The conjunctive four-block search design may also have excluded relevant STEM career studies that used adjacent terminology or did not include at least one term from each block in the database-specific searchable fields. Therefore, the corpus should be understood as a focused representation of a predefined conceptual configuration rather than a comprehensive map of all STEM career-choice research. Because the three psychosocial constructs were placed within the same alternative search block, each publication was not required to address all of them simultaneously. The findings therefore represent their relative positions within the selected corpus rather than their importance across the entire field. Future research could compare broader, construct-specific, or differently configured corpora.

Third, the conceptual structure analyses depended on the author-provided and database-indexed keyword information contained in the All Keywords field. Therefore, the keyword-based maps depended on the availability, consistency, and specificity of keyword metadata rather than on a full-text coding of each publication. Although spelling, hyphenation, singular–plural differences, and non-substantive terms were cleaned and normalized, keyword harmonization involves analytical judgment. Even though the normalization procedure was conducted systematically, decisions about synonym merging, preferred terms, and the removal of indexing terms may have introduced a degree of researcher subjectivity. Some related terms may have remained separate, while certain distinctions may have been reduced through merging. Moreover, bibliometric co-occurrence and thematic mapping reveal how concepts are represented together in the literature but do not establish causal relationships or test the pathways proposed by Social Cognitive Career Theory. SCCT was therefore used only as an organizing interpretive lens. Full-text reviews, qualitative analyses, and longitudinal or mixed-method studies are needed to examine the empirical relationships among gender, identity, self-efficacy, self-concept, and STEM career choice.

Finally, the temporal and collaboration findings require caution. The year 2026 was incomplete at the time of data collection, and indexing delays may have affected the number of recent publications. The lower 2026 output should not be interpreted as a decline, and the exploratory life-cycle model should not be treated as a definitive forecast. Citation-related indicators should also be interpreted cautiously because they may be affected by publication age, journal visibility, database indexing, language, and field-specific citation practices rather than reflecting scholarly influence alone. Country frequencies were based on author-affiliation occurrences rather than unique publication counts, while SCP–MCP indicators do not capture every dimension of international collaboration. Reproducibility may also be affected by later database updates and licensing restrictions.

## Data Availability

Publicly available datasets were analyzed in this study. The data used in this study were obtained from the Web of Science and Scopus databases. However, the dataset generated and analyzed during the current study is available from the corresponding author upon request.
